# Universal health coverage, oral health, equity and personal responsibility

**DOI:** 10.2471/BLT.19.247288

**Published:** 2020-09-03

**Authors:** Tim T Wang, Manu Raj Mathur, Harald Schmidt

**Affiliations:** a University of Pennsylvania, 423 Guardian Drive, Philadelphia, Pennsylvania 19104–4884, United States of America.; b Public Health Foundation of India, New Delhi, India.

Universal health coverage (UHC) is broadly defined as all individuals having access to needed health services without financial hardship.^1^ Over the last decade, the push for UHC has gained considerable momentum, having become a priority area in reaching the sustainable development goals (SDGs) by 2030. Initial evaluations indicate progress in important areas such as coverage of human immunodeficiency virus, tuberculosis and malaria services.^1^ Oral health, by contrast, has been largely absent from the UHC discussion, and limited progress has been made in addressing oral diseases around the world over the last twenty years.^2^

In many countries, oral health is deemed low priority and attributed to individual, rather than social responsibility. However, a few countries working towards UHC include dental services for some or all population groups, suggesting that the exclusion of oral health from UHC is no conceptual inevitability. Failing to broaden UHC to encompass oral health risks undermining systemic health outcomes and exacerbating health disparities. As countries plan and align strategies towards UHC, reviewing whether excluding oral health is compatible with the overall goals of UHC is necessary.

To monitor progress towards UHC, the World Health Organization (WHO) and the World Bank use 16 tracer indicators in four categories: (i) reproductive, maternal, and child health; (ii) infectious diseases; (iii) noncommunicable diseases; and (iv) service capacity and access.^1^ Though poor oral health is recognized as a noncommunicable disease, it was excluded from being an indicator for the health-related SDG, which aims to broadly improve health outcomes.

## Omission from UHC 

Historically, dental services have been treated as nonessential parts of health systems and relegated to personal responsibility. In the United Kingdom of Great Britain and Northern Ireland, a single-payer system, dental services were initially covered by the National Health Service. However, the government instituted a co-payment for services in 1952 as a cost-saving measure.^3^ Cutting dental coverage in view of limited budgets suggests that dental care was a discretionary benefit that, unlike other medical services, need not be unconditionally provided under the UHC of the national health system.

In Canada, another country noted for comprehensive health coverage, dental services are incompletely covered by Medicare. Coverage varies by provinces and differentially extends to groups such as indigenous peoples, veterans, low-income families and other vulnerable populations.^4^ The status of dental coverage can be attributed at least in part to the belief that maintaining good oral health and seeking treatment are up to the individual. Providing the blueprint for the programme, the Royal Commission on Health Services reasoned that “personal hygiene [and] balanced diets [are] under the control of the individual [and s/he] must assume responsibility for wise and prudent use of health services [..] including regular dental examinations.”^4^

The United States of America, which does not offer universal health care, mostly excluded dental benefits when it established Medicaid and Medicare for vulnerable populations.^5^ Despite the prominent expansion of the social safety net for health care, public dental coverage was only initially provided to children from low-income families and only gradually extended to low-income adults voluntarily by some states. These examples illustrate that neglect of oral health is a common theme in many high-income countries regardless of their health care model.

## Role of oral health 

Most high-income countries currently implement UHC with partial dental coverage, resulting in disparities in access to care and oral health outcomes. A 2010 survey of 29 Organisation for Economic Cooperation and Development countries found that only five (Austria, Mexico, Poland, Spain and Turkey) covered the full cost of dental care and six (Belgium, Finland, Germany, Iceland, Japan and United Kingdom) covered 76–99% of the costs.^6^ Three countries (Luxembourg, Republic of Korea and Slovakia) covered 50–75%. Nine countries (Czech Republic, Denmark, France, Greece, Hungary, Italy, Netherlands, Portugal and Sweden) covered less than half of dental costs, and six (Australia, Canada, Ireland, New Zealand, Norway and Switzerland) did not cover any. Instead of integrating dental care into UHC, many countries provide coverage to certain subgroups, commonly children and low-income individuals.

Dental services are similarly relegated to the sidelines of UHC in low- and middle-income countries. In fact, the World Bank’s Universal Health Coverage Study Series, which documents and analyses low-income countries’ paths towards UHC, omits oral health from its monitoring of benefit packages.^7^ Nevertheless, a few countries have made strides towards aligning dental care with UHC initiatives. For example, Brazil integrated dental and primary medical care through the nation policy *Brasil Sorridente* (Smiling Brazil) in 2004.^8^ However, most low- and middle-income countries do not have UHC initiatives that integrate universal oral health programmes.

## Effect of neglect

Though mostly preventable, oral diseases are rampant in countries of all levels of development, affecting 60–90% of children and most adults.^2^ Untreated oral diseases can have debilitating to fatal consequences. In addition to tooth decay and gum disease, oral pathologies include oral and oropharyngeal cancers, microbial infections and tooth abscesses. Poor oral health also lowers quality of life by limiting the ability to chew and reducing self-esteem.^9^ Moreover, oral diseases share common risk factors, including tobacco, alcohol, diet and stress, with other major noncommunicable diseases such as cardiovascular disease and diabetes.^10^ As such, excluding oral health from universal health schemes jeopardizes long-term population health outcomes far beyond the mouth. Furthermore, this neglect perpetuates the notion that oral health is conceptually distinct from systemic health.

Neglecting oral health also has negative consequences for health equity. The fact that most oral diseases can be prevented with early intervention may underlie the emphasis on personal responsibility in maintaining good oral health. But this interpretation supposes that across population groups, everyone has equal opportunity to health-care access, and overlooks a wide range of socioeconomic determinants that impact oral health, sometimes even before birth. In 2016, the World Dental Federation adopted a new definition of oral health and identified five driving determinants for oral health outcomes: genetic and biological factors, social environment, physical environment, health behaviours and access to care.^9^ While healthy behaviours are essential to maintaining good oral health, focusing solely on personal responsibility is not the answer.

Globally, dental diseases disproportionately afflict low-income populations.^10^ Oral diseases can reduce employment opportunities and limit upward social mobility, thus reinforcing social inequalities beyond health. Oral health disparities are particularly stark in high-income countries, such as the United States, where low-income adults face structural and economic obstacles to access basic preventative and restorative dental services. In low- and middle-income countries, dental treatment has been found to be a significant source of catastrophic health expenditure.^11^ On the other hand, European countries that offer public dental coverage were found to have lower inequalities in the usage of dental care.^12^ As such, UHC inclusive of dental services can help promote equity in terms of access to care and oral health outcomes that have individual, societal and health-care system benefits.

## Addressing the gaps

Oral health is an essential component of well-being that has significant effects on systemic health and health equity. While personal responsibility does play a role, full recognition of the social determinants of health and people’s opportunities of choice makes singling out dental care conceptually implausible. As such, countries working towards UHC that do not yet include dental services should reconsider the adequacy of their strategy. WHO could include oral health in its next iteration of its Core Health Indicators, and the United Nations and the World Bank could integrate oral health in their monitoring frameworks for assessing UHC progress.

Because they share many common risk factors, oral diseases should be integrated into mainstream efforts to reduce noncommunicable diseases. A basic package of dental services can be incorporated into primary health care initiatives to leverage pre-existing medical infrastructure to reduce the burden of oral diseases. Such services would boost routine oral health surveillance and dental service coverage and would dispel the dangerous precedent of isolating oral from systemic health. Heightened policy attention towards oral health and noncommunicable diseases can enhance data collection and support analyses of dental services and relevant outcomes. Better data are critical for improving access to care and designing appropriate public health measures in areas such as water fluoridation, workforce training and integrating oral examinations into primary medical care.

In combination with policy efforts, engaging the local population is key to integrating oral health into UHC. Empowered communities aware of the integral role of oral health in overall well-being can demand for the right to access dental services, particularly in the context of efforts towards achieving UHC. Finally, oral health has been found to be a key indicator of inequity in other social determinants, such as education and economic status.^10^ Therefore, improving oral health is consistent with the intentions of the broader set of SDGs, as it helps make progress towards the health-related SDG and SDG 10, which aims to reduce socioeconomic disparities.

To date, the progress towards UHC has been encouraging, but the patchy approach towards including dental services still stands in the way of truly comprehensive UHC and risks jeopardizing health improvements of vulnerable populations due to misconceived notions of personal responsibility.

## References

[1] Primary health care on the road to universal health coverage: 2019 monitoring report. Geneva: World Health Organization; 2019. Available from: https://www.who.int/healthinfo/universal_health_coverage/report/uhc_report_2019.pdf?ua=1 [cited 2020 Jun 2].

[2] Kassebaum NJ, Bernabé E, Dahiya M, Bhandari B, Murray CJL, Marcenes W. Global burden of untreated caries: a systematic review and metaregression. J Dent Res. 2015 5;94(5):650–8. 10.1177/0022034515573272 25740856

[3] The National Health Service - Management in the 1950s [internet]. London: The National Archives of the UK. Available from: http://www.nationalarchives.gov.uk/cabinetpapers/alevelstudies/management-1950.htm [cited 2018 Aug 9].

[4] Quiñonez C. Why was dental care excluded from Canadian Medicare? Toronto: University of Toronto, Network for Canadian Oral Health Research; 2013 Available from: http://www.ncohr-rcrsb.ca/knowledge-sharing/working-paper-series/content/lay-quinonez.pdf [cited 2018 Sep 10].

[5] Cohen W, Ball R. Social Security Amendments of 1965: Summary and Legislative History. Soc Secur Bull. 1965 9;28(9):3-21

[6] Paris V, Devaux M, Wei L. Health systems institutional characteristics: a survey of 29 OECD countries. Report No.50. Paris: Organisation for Economic Co-operation and Development; 2010. Available from: https://www.oecd-ilibrary.org/social-issues-migration-health/health-systems-institutional-characteristics_5kmfxfq9qbnr-en [cited 2018 Jul 9].

[7] Going Universal - How 24 countries are implementing universal health coverage reforms from the bottom up. Washington, DC: World Bank; 2015. Available from: http://www.worldbank.org/en/topic/universalhealthcoverage/publication/going-universal-how-24-countries-are-implementing-universal-health-coverage-reforms-from-bottom-up [cited 2018 Aug 1].

[8] Pucca GA Jr, Gabriel M, de Araujo ME, de Almeida FCS. Ten years of a national oral health policy in brazil: innovation, boldness, and numerous challenges. J Dent Res. 2015 10;94(10):1333–7. 10.1177/0022034515599979 26316461

[9] Glick M, Williams DM, Kleinman DV, Vujicic M, Watt RG, Weyant RJ. A new definition for oral health developed by the FDI World Dental Federation opens the door to a universal definition of oral health. J Public Health Dent. 2017 12;77(1):3–5. 10.1111/jphd.12213 28276588

[10] Peres MA, Macpherson LMD, Weyant RJ, Daly B, Venturelli R, Mathur MR, et al. Oral diseases: a global public health challenge. Lancet. 2019 7 20;394(10194):249–60. 10.1016/S0140-6736(19)31146-8 31327369

[11] Bernabé E, Masood M, Vujicic M. The impact of out-of-pocket payments for dental care on household finances in low and middle income countries. BMC Public Health. 2017 1 23;17(1):109. 10.1186/s12889-017-4042-0 28114967 PMC5260123

[12] Listl S. Countries with public dental care coverage have lower social inequalities in the use of dental services than countries without such coverage. J Evid Based Dent Pract. 2015 3;15(1):41–2. 10.1016/j.jebdp.2014.12.001 25666584

